# Om chanting modulates the processing of negative stimuli: Behavioral and electrophysiological evidence

**DOI:** 10.3389/fpsyg.2022.943243

**Published:** 2022-10-13

**Authors:** Ziyu Zhang, Yanqin Peng, Tingji Chen

**Affiliations:** Department of Psychology, School of Education, Soochow University, Suzhou, China

**Keywords:** Om chanting, emotional processing, event-related potential, P1, late positive potential

## Abstract

Previous studies have suggested that Om chanting, a type of meditation, can relieve individuals' negative emotions. However, the dynamic aspects of neural processes in the processing of the negative stimuli while Om chanting are still unclear. In this study, we recruited 33 healthy undergraduate students without meditation experience and recorded event-related potentials (ERPs) to unpleasant and neutral images when they performed Om chanting and viewing task. The behavioral results showed that the unpleasant images were rated as less unpleasant and arousing in the condition of Om chanting than while passive viewing, and the rates were not different between the two conditions for the neutral images. Analyses of the ERP responses to the emotional stimuli revealed that Om chanting decreased P1 and late positive potential (LPP) amplitudes for the neutral images but not for the unpleasant images. We speculated that Om chanting might reduce vigilance to the neutral stimuli, whereas for the negative stimuli, they automatically captured all available attentional resources and led to a failure in observing the regulating effect of Om chanting. These observations suggest that Om chanting modulates individuals' affective evaluations to the negative stimuli and alters early visual and late neural processing of the stimuli.

## Introduction

Om chanting, originated in Hinduism, is a meditation that involves constantly repeating three syllables (A, U, and M) silently or aloud while passively disregarding any internal or external distractions (Lynch et al., 2018). In this process, the particular body vibration that the three phonemes induce is believed to calm and focus the mind and body without the need for intense concentrative efforts (Braboszcz et al., 2010).

Previous literature has demonstrated a positive impact of Om chanting on relaxation. For example, by measuring the rate of respiration (RR) and heart rate (HR), a study showed that 20 days of Om chanting practice decreased RR and HR values and led to a state of relaxation (Telles et al., 1998). Harne and Hiwale (2018) recorded 30-min resting electroencephalogram (EEG) signals of participants before and after performing loud Om chanting and found a larger theta power after than before Om chanting. The enhancement of theta power has been suggested to reflect relaxation (see also, Jacobs and Friedman, 2004). Om chanting has also been suggested to reduce participants' negative affective states. A previous study has shown that after practicing Om chanting for 4 weeks for 20 min a day and 6 days per week, bus drivers' anxiety level, assessed by Hamilton anxiety rating scale (HAM-A), has significantly reduced, as compared with bus drivers who did not practice Om chanting (Rankhambe and Pande, 2020). Similarly, after 6 months of Om chanting, depression, anxiety, and stress of elderly women with hypertension have been significantly decreased, as compared with the control group (Amin et al., 2016). These research studies suggest that short-time Om chanting training can relieve negative affective states.

Prior neuroimaging studies have examined brain mechanisms while Om chanting. An fMRI study assessed neurohemodynamic correlates of loud Om chanting and found that as compared with the resting brain state, Om chanting led to a significant deactivation in the amygdala, whereas neither activation nor deactivation was observed in the condition of chanting the sound “ssss” (Kalyani et al., 2011). The amygdala has been demonstrated to play an important role in emotional processing, particularly in the process of negative emotions (Hamann et al., 2002; Baas et al., 2004). A possible reason for the deactivation in amygdala is because Om chanting activates brain regions that involve emotion regulation, such as prefrontal cortex (PFC). This speculation is supported by a more recent fNIRS study (Deepeshwar et al., 2015). In this study, the authors evaluated relative hemodynamic changes in the PFC during a color–word Stroop task after a 20-min Om chanting, and the results showed that Om chanting enhanced performance and efficiency in task; meanwhile, oxygenation levels increased in the PFC while Om chanting (Deepeshwar et al., 2015). In conclusion, neuroimaging studies have revealed that brain regions related to emotion and regulation processing are involved in Om chanting. However, the dynamic aspects of neural processes in processing of negative stimuli while Om chanting are still unclear. Particularly, it remains unknown at which stage of processing Om chanting starts to exert its influence on emotional processing. Does it occur at an early or late processing stage? The high temporal resolution of event-related potentials (ERPs), which provide a continuous time window on neural processes during stimulus presentation, may shed light on this question.

In the present study, we investigated the effects of Om chanting on emotional processing by measuring ERP responses to unpleasant and neutral images. Based on the previous studies, we focused on two ERP components elicited by emotional pictures in the task. Numerous studies have suggested that ERP indices of affective processing are related to modulations in attentional regulatory processes (e.g., P1) occurring at an early stage and cognitive regulatory processes (e.g., late positive potential, LPP) occurring at a late stage (e.g., Hajcak et al., 2010). P1 originating in the extrastriate visual cortex peaks at 80–150 ms poststimulus (Di Russo et al., 2005). Studies have shown that the P1 component is sensitive to attention allocation and modulated by the affective valence of emotional stimuli (e.g., Smith et al., 2003; Holmes et al., 2009). Importantly, the P1 amplitude has been found to be significantly attenuated during tasks involving inward attention (i.e., generation of inner speech and performance of visual imagery) as compared with tasks involving external attention (Villena-González et al., 2016). In the late processing stage, a component termed LPP is a positive-going slow wave that is maximal at central parietal sites. It occurs at 300 ms after stimulus presentation and can last for several seconds (Schupp et al., 2006; Thiruchselvam et al., 2011). According to the previous studies, the LPP component not only is modulated by the affective valence of the stimuli, with larger LPP elicited by the emotional than neutral stimuli (Hajcak et al., 2009), but also reflects elaborated processing related to the significance and meaning of stimuli (MacNamara et al., 2009). For example, Foti and Hajcak (2008) presented participants with unpleasant images preceded by either more negative or more neutral descriptions. The results showed that the LPP to the unpleasant images was largely reduced when preceded by the neutral vs. negative descriptions. This finding suggests that the meaning in the descriptions which lead to reappraisal processing can modulate the LPP responses to the affective stimuli. By investigating the components of P1 and LPP, the aim of the current study was to shed light on the mechanisms underlying the effect of Om chanting on the emotional processing.

According to the findings which revealed the modulation of P1 and LPP by the affective valence (Schupp et al., 2006; Olofsson et al., 2008), we hypothesized that larger P1 and LPP amplitudes would be observed for the unpleasant vs. neutral images (hypothesis I). As studies have shown that meditation can reduce the amplitude of P1 and LPP elicited by unpleasant stimuli (Sobolewski et al., 2011; Zhang et al., 2019), we predicted that the Om chanting, a type of meditation, would decrease P1 and LPP amplitudes to unpleasant images relative to passive viewing (hypothesis II).

## Method

### Participants

The study recruited 33 undergraduate students (22 female individuals; Mage = 19.73, SD age = 1.24) through advertisements at Soochow University in Suzhou, China. All participants were right-handed, had normal or corrected-to-normal vision, and reported no history of psychiatric or neurological disease. The participants reported that they were not acquainted with meditation or had little meditation experience. All experimental procedures were approved by the Soochow University Human Ethics Committee. The participants provided written informed consent prior to the experiment and were paid 45 yuans (equivalent of 7.07 dollars) for their participation.

### Stimuli

The stimuli were 112 image stimuli (56 negative and 56 neutral) selected from the Chinese Affective Picture System (CAPS) (Bai et al., 2005) in which a set of 825 images were rated from 1 to 9 for their valence and arousal (1 = very unpleasant/calming, 9 = very pleasant/arousing) by 46 individuals. According to the ratings, the selected negative images (valence: 2.18 ± 0.45, *M* ± *SD*; arousal: 5.50 ± 0.24) were rated significantly more negative and more arousing than the neutral images (valence: 5.14 ± 0.18; arousal: 4.41 ± 0.29, both *ps* < 0.001).

### Design and procedure

After the participants provided informed consent upon arrival, they were attached with the EEG sensors. Before the actual experiment, the participants were instructed to practice Om chanting for 3 min. They were guided to keep spine erect and recite A, U, and M loudly and perceive the vibration inside the body, and then to repeat silently while imagining the flow of air from the abdominal cavity to the thoracic cavity and then to the oral cavity. After the participants were familiar with the procedure of chanting, we started the EEG recording and then the actual experiment began. The participants were required to perform a silent chanting in their minds avoiding any movement of the lips in the actual experiment.

**Figure 1:**
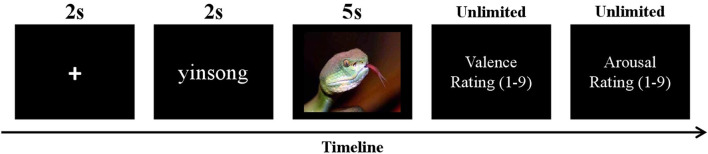
A single trial in the task.

The present experiment had a 2 (trial type: passive viewing, Om chanting) × 2 (stimulus valence: negative, neutral) within-subject design, including four blocks with 28 trials of emotional images in each. Each block contained equal numbers of stimuli of four conditions. Overall, the experiment included 112 trials with each stimulus presented one time. The order of the presented trials was randomized. In each trial, a fixation cross was first presented in the center of the screen for 2,000 ms. Then, Chinese words of “guankan” (viewing) or “yinsong” (Om chanting) which instructed the participants the task in the current trial appeared for 2,000 ms. Then an emotional image was displayed for 5,000 ms, and the participants were required to carry out the task (viewing or Om chanting) until the image disappeared. In the Om chanting condition, the participants were asked to chant Om silently (without any lip movements) when viewing images. The viewing condition required the participants to passively view the pictures without any form of chanting. After the offset of the image, the participants evaluated the affective valence and arousal of the image on the 9-point scales (1 = very unpleasant/calming, 9 = very pleasant/arousing). A sequence of events on a single trial in the task is illustrated in Figure 1.

To assess whether the participants have followed the instruction and performed Om chanting, they were requested to complete a questionnaire that inquired on the extent to which they performed the Om chanting whenever they were instructed to do so on a 1–7 Likert scale ranging from 1 (never) to 7 (completely).

The experiment was conducted in a dimly lit and sound-attenuated booth. E-prime 2.0 was used to record behavioral data and present all instructions and prompts on a 27-inch (2,560 × 1,440) computer screen with 144 Hz refresh rate. Each image was displayed in the center of the monitor with a viewing distance of 100 cm.

### Data collection and analysis

Electroencephalogram (EEG) data were gathered with 64 Ag/AgCl electrodes attached in a BrainCap (Brain Product, Germany) according to the International 10–20 System. Horizontal eye movements were detected by two extra bipolar electrodes placed at the outer canthi of the eyes (horizontal electrooculogram, HEOG), and vertical eye movements were detected by another two electrodes, placed above and below the left eye (vertical electrooculogram, VEOG). The impedances of all electrodes were kept below 5 k*Ω* during data recording. The raw EEG and EOG signals were amplified with a gain of 10,000, filtered with an amplifier bandpass of 0.05–100 Hz, and were digitized with a sampling rate of 1,000 Hz.

Offline, the EEG data were processed and analyzed with the EEGLab (http://erpinfo.org/erplab/) and ERPLab (http://sccn.ucsd.edu/eeglab/) toolbox on the MATLABTM platform (MathWorks Inc. USA). In the pre-processing stage, the data were resampled at 500 Hz. The signals were converted to an average mastoid reference and bandpass-filtered at 0.1–30 Hz with a 24-dB/oct slope. An independent component analysis (ICA) was run to detect and correct ocular artifacts. The EEG signals were segmented for each trial, beginning 200 ms before and 1,200 ms after the stimulus onset. The pre-stimulus interval (i.e., 200 ms) was applied for baseline correction. Epochs were excluded from averaging if they contained activity exceeding ± 100 μV at any site. In total, 5.36% trials were excluded from further analysis. The average accepted trials in each condition are presented in Supplementary Table 1. Based on the accepted trials, average waveforms in each experimental condition were calculated for each participant.

Selection of the electrode sites and time windows was based on visual inspection of the averaged waveforms and the findings from previous studies (Brown et al., 2012; Schindler and Straube, 2020; Lutz et al., 2021; Schindler et al., 2021). According to the previous research, P1 amplitude was defined as the mean amplitude within an 85–125-ms window, LPP amplitude was defined as a 400- to 1200-ms window following the picture onset at the parietooccipital site group (P3, PZ, P4, PO7, POZ, PO8, O1, O2, Oz).

### Data analysis

Two-way repeated measures ANOVAs with 2 (trial type: passive viewing, Om chanting) × 2 (stimulus valence: negative, neutral) were performed for both behavioral (valence and arousal rating) and ERP data (mean amplitudes of P1, LPP). All statistical analyses were performed using the SPSS package. The Greenhouse–Geisser correction was applied, when appropriate. For clarity, uncorrected degrees of freedom are reported.

## Results

The participants were requested to complete a questionnaire at the end of the experiment to determine whether they performed Om chanting according to the instruction. The results showed that the participants seriously performed Om chanting during the experiment (*M* = 5.24, *SD* = 1.20).

### Behavioral data

For the valence rating, the main effect of trial type was not significant, *F*_(1, 32)_ = 3.61, *p* = 0.07, $\eta_{p}^{2}$ = 0.10. A two-way ANOVA showed a significant main effect of stimulus valence, *F*_(1, 32)_ = 299.41, *p* < 0.001, $\eta_{p}^{2}$ = 0.90. Negative images (2.69 ± 0.71) were rated as more unpleasant than neutral images (4.78 ± 0.65). The analysis showed a significant interaction of trial type × stimulus valence levels, *F*_(1, 32)_ = 12.40, *p* < 0.05, $\eta_{p}^{2}$ = 0.28. Planned comparison showed that negative images were rated less unpleasant under the conditions of Om chanting (2.80 ± 0.73) than while viewing (2.58 ± 0.68), *t*_(32)_ = 3.17, *p* < 0.05, *d* = 0.31. No differences between the conditions of Om chanting (4.74 ± 0.65) and viewing (4.81 ± 0.65) were found when rating the neutral images, *t*_(32)_ = 1.65, *p* = 0.11, *d* = 0.11.

For the arousal rating, a two-way ANOVA showed a significant main effect of trial type, *F*_(1, 32)_ = 5.06, *p* < 0.05, $\eta_{p}^{2}$ = 0.14. Images were rated as less arousing on the conditions of Om chanting (4.17 ± 1.50) vs. viewing (4.33 ± 1.50). Moreover, there was a significant main effect of stimulus valence, *F*_(1, 32)_ = 64.02, *p* < 0.001, $\eta_{p}^{2}$ = 0.67. Negative images (5.56 ± 1.58) were rated as more arousing than neutral images (2.95 ± 1.42). The interaction between trial type and stimulus valence was not significant, *F*_(1, 32)_ = 0.84, *p* = 0.37, $\eta_{p}^{2}$ = 0.03.

### ERP data

#### P1 amplitude

**Figure 2:**
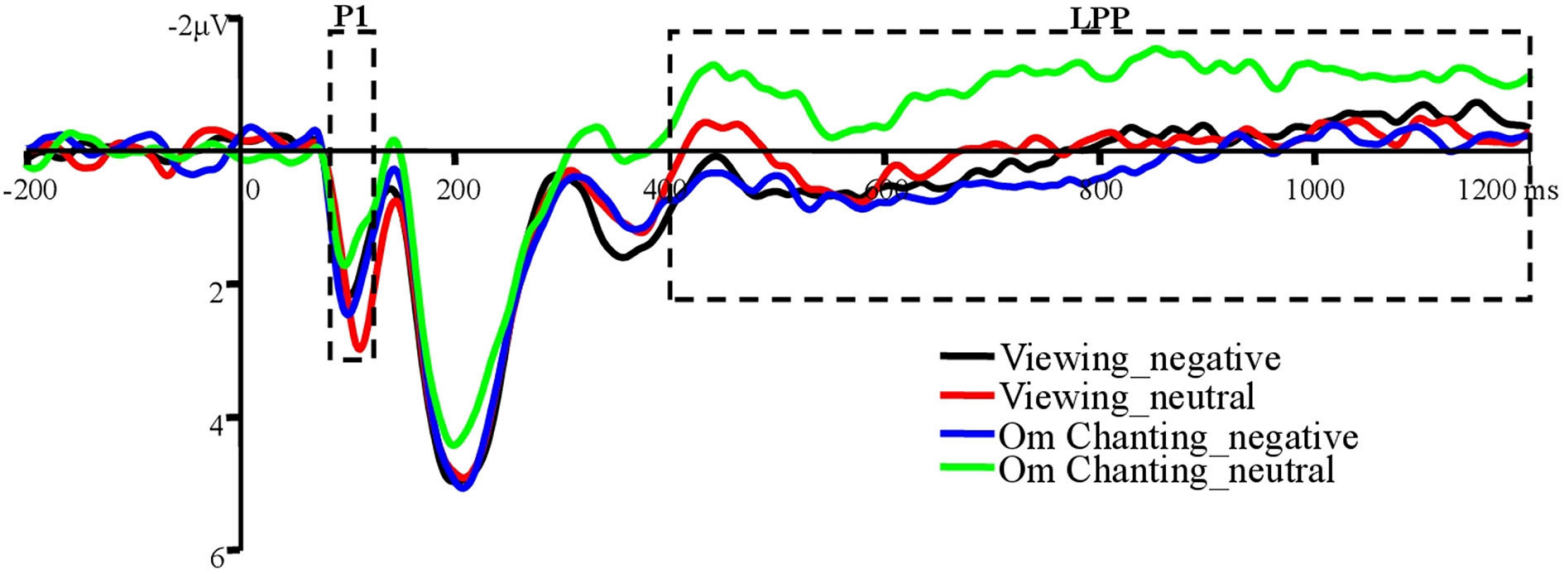
Grand average ERPs of P1 and LPP to unpleasant and neutral images at the parietooccipital site group (P3, PZ, P4, PO7, POZ, PO8, O1, O2, and Oz).

There was no main effect of trial type, *F*_(1, 32)_ = 1.36, *p* = 0.25, $\eta_{p}^{2}$ = 0.04. No significant main effect of stimulus valence was obtained, *F*_(1, 32)_ = 0.55, *p* = 0.46, $\eta_{p}^{2}$ = 0.02. The interaction between trial type and stimulus valence was significant, *F*_(1, 32)_ = 20.66, *p* < 0.001, $\eta_{p}^{2}$ = 0.39. Planned comparison showed that neutral images elicited lower P1 amplitudes under the conditions of Om chanting (1.29 ± 2.21) than while viewing (2.08 ± 2.36), *t*_(32)_ = −3.31, *p* < 0.05, *d* = 0.35. No differences between the conditions of Om chanting (1.92 ± 2.34) and viewing (1.66 ± 2.44) were found when negative images were presented, *t*_(32)_ = 0.98, *p* = 0.34, *d* = 0.11 (Figure 2).

#### LPP amplitude

There was no main effect of trial type, *F*_(1, 32)_ = 1.81, *p* = 0.19, $\eta_{p}^{2}$ = 0.05. A significant main effect of stimulus valence was obtained, *F*_(1, 32)_ = 11.48, *p* < 0.05, $\eta_{p}^{2}$ = 0.26. Negative images (0.14 ± 2.45) elicited greater LPP amplitudes than neutral images (−0.54 ± 1.94). The interaction between trial type and stimulus valence was significant, *F*_(1, 32)_ = 10.30, *p* < 0.05, $\eta_{p}^{2}$ = 0.24. Planned comparison showed that neutral images elicited lower LPP amplitudes under the conditions of Om chanting (−1.02 ± 2.02) than under viewing (−0.05 ± 2.36), *t*_(32)_ = −2.73, *p* < 0.05, *d* = 0.44. No differences between the conditions of Om chanting (0.26 ± 2.22) and viewing (0.02 ± 2.93) were found when negative images were presented, *t*_(32)_ = 0.78, *p* = 0.44, *d* = 0.09 (Figure 2).

## Discussion

By measuring ERP responses to unpleasant and neutral images at the experiment, the present study investigated the effects of Om chanting on emotional processing. The behavioral results showed that Om chanting reduced the unpleasant and arousal ratings of the negative images. Furthermore, the ERP results indicated that Om chanting attenuated processing of neutral images at both the early and late stages.

An important finding in the present study was that the behavioral rating results revealed an interaction between stimulus valence and trial type. Specifically, negative stimuli were rated as less unpleasant and less arousing in the condition of Om chanting than under passive viewing, whereas for the neutral stimuli, the rates were not different between the conditions of Om chanting and passive viewing. These results indicate that Om chanting, a type of meditation, can regulate and improve individuals' emotion when process negative stimuli and lead to a state of relaxation, and thus modulate the affective ratings on a behavioral level. Zhang et al. (2019) found that breath-focused mindfulness, a type of meditation which requires participants to focus on internal feelings, produced lower arousal ratings to the affective images than passive viewing. The authors suggested that the meditation allowed the participants to concentrate on the unfolding of experience moment by moment without attaching any evaluative valence to the self-referential processing experience (see also, Kabat-Zinn, 2003; Cahn and Polich, 2006). This may also be the explanation for the present findings. Consistently, previous fMRI studies also suggested that Om chanting activated the PFC, a brain region which involves the emotion regulation and attenuated the responses in the amygdala, which plays an important role in the process of negative emotions.

Unexpectedly, the ERP results showed that as compared with passive viewing, Om chanting performed by the participants decreased P1 and LPP amplitudes to the neutral images, whereas for the unpleasant images, the two components showed no differences between the Om chanting and viewing condition. These results are contrary to our hypothesis. One possible reason is that Om chanting may reduce vigilance and intensity and thus allocated less attentional resources, at both early visual and late more elaborated processing, to the neutral stimuli. However, for the negative stimuli, possibly, the failure in observing the regulating effect of Om chanting may have been due to a kind of floor effect on the P1 and LPP elicited by highly negative stimuli (Moser et al., 2006; Krompinger et al., 2008); that is, highly negative stimuli may automatically capture all available attentional resources (for a review, see Hajcak et al., 2010) so that the neural responses to them were difficult to be modulated by an instant and brief Om chanting. This result is consistent with the finding reported in the previous ERP studies that practitioners without meditation experience fail to use meditation to regulate their automatic responses and judgments to the negative stimuli. For example, Eddy et al. (2015) used a within-subject repeated measures design to examine whether a brief mindfulness induction could modulate brain potentials during emotional processing. The findings indicated that compared with the control condition, brief mindfulness did not produce ERP (P300 and LPP components) differences while processing of emotional images. However, the aforementioned explanations might be speculative and still need to be verified in future studies. It is noted that in the study by Eddy et al. (2015), they adopted brief mindfulness, a meditation that is quite different from Om chanting in terms of form and content (Dahl et al., 2015; Harne et al., 2019), and they did not require the participants to evaluate the emotional dimensions of the images in each trial. These may make the comparisons less accurate and comprehensive. One might speculate why the behavioral ratings but not the ERP responses to the unpleasant images were modulated by Om chanting. It should be noted that even though P1 and LPP components can be modulated by the emotional valence, these two components are not direct indexes to assess or predict individuals' responses to emotional valence (Olofsson et al., 2008). Therefore, it may not be appropriate to directly compare the behavioral rating results with the ERP findings. In addition, consistent with numbers of previous studies regarding the ERP responses to emotional stimuli (Foti and Hajcak, 2008; Olofsson et al., 2008), the current ERP results demonstrated a modulation of LPP by the emotional valence, showing greater LPP amplitudes elicited by the unpleasant images than the neutral images in the experiment. The main effect of stimulus valence on LPP might serve as evidence to support a reliability of the present data.

The present study has some limitations that should be pointed out. First, the trial number included in the ERP analysis was relatively small, with only 28 trials in each condition. Previously, studies have recommended at least 30 trials per condition in order to obtain reliable measures for ERP components, depending on the factors such as the sample size, the anticipated effect magnitude, and the within- or between-subject design (e.g., Thigpen et al., 2017; Boudewyn et al., 2018). Thus, future studies could increase the number of trials to further examine the effect of Om chanting on emotional processing. Second, the present study included only neutral and negatively valanced stimuli, but not positively valanced stimuli, such as pleasant images. Therefore, it remains unclear whether or how Om chanting exerts the influence on the positive stimuli. This question can be addressed in the future studies. Third, the emotional stimuli used in the present study were from a Chinese affective picture database, that is, CAPS, in which a relatively small number of participants recruited for the stimulus evaluations may limit the reliability and validity of the rating data. Thus, in future studies, this could be improved by conducting a pilot study which includes a sufficient number of participants to assess the selected stimuli prior to the actual experiment. Finally, the present study included a condition of passive viewing as a control condition to compare with the condition of silent Om chanting. However, it should be noted that the processes of vocalization and speech production that Om chanting may involve are apparently different from the processes of passive viewing. Vocalization is a laryngeal motor behavior that involves highly specialized coordination of laryngeal and respiratory neuromuscular control (Loucks et al., 2007). Speech production is the process of organizing communication intentions, activating concepts, and extracting lexical, syntactic, and phonological information to controlling the articulatory organs to produce sounds, and it involves more top-down processing than passive viewing (Dell, 1986; Ackermann et al., 2004). Therefore, we suggest that future studies could adopt a control condition that involves a loud or silent repetition of a group of words, phrases, or syllables, for example, “sssss” or “one” (Telles et al., 1998; Kalyani et al., 2011), to better clarify the effect of Om chanting on emotional processing.

## Conclusion

In the present study, we examined the behavioral and ERP responses to unpleasant and neutral images while performing Om chanting. The behavioral results indicated an effect of Om chanting on regulating the emotional responses, showing reduced unpleasant and arousal ratings for the negative stimuli in the condition of Om chanting. Furthermore, analyses of the ERP data showed modulation of P1 and LPP responses to the neutral stimuli caused by Om chanting. The findings provide preliminary evidence that Om chanting modulates individuals' affective evaluations to the emotional stimuli and alters early visual and late neural processing of the stimuli, and they may help clarify the mechanisms of the role of Om chanting in emotional processing.

## Data availability statement

The raw data supporting the conclusions of this article will be made available by the authors, without undue reservation, to any qualified researcher.

## Ethics statement

The studies involving human participants were reviewed and approved by Institutional Review Board (or Ethics Committee) of Soochow University. The patients/participants provided their written informed consent to participate in this study.

## Author contributions

This work is the result of collaboration between ZZ, YP, and TC. All authors have equally contributed, reviewed, improved the manuscript, and read and agreed to the published version of the manuscript.

## Funding

This research was supported by the Natural Science Foundation of Jiangsu Province (Grant No. BK20200863 to TC) and Humanities and Social Sciences Planning Fund of the Chinese Ministry of Education (Grant No. 20YJA190007 to YP).

## Conflict of interest

The authors declare that the research was conducted in the absence of any commercial or financial relationships that could be construed as a potential conflict of interest.

## Publisher's note

All claims expressed in this article are solely those of the authors and do not necessarily represent those of their affiliated organizations, or those of the publisher, the editors and the reviewers. Any product that may be evaluated in this article, or claim that may be made by its manufacturer, is not guaranteed or endorsed by the publisher.
