# Supplementary material for: Om chanting modulates the processing of negative stimuli: Behavioral and electrophysiological evidence
Source: Front Psychol. 2022 Oct 13;13:943243. doi: 10.3389/fpsyg.2022.943243 (PMC9606574; doi:10.3389/fpsyg.2022.943243)
Supplement: Supplementary file 1 [file Table_1.DOCX]

Supplementary Material

# Supplementary Table

**Supplementary Table S1.** The average accepted trials in each condition (*M* ± *SD*)

|  | **Negative** | **Neutral** |
| --- | --- | --- |
| **Om chanting** | 26.39 ± 3.67 | 26.67 ± 3.94 |
| **Viewing** | 26.52 ± 3.18 | 26.42 ± 3.27 |
